# Editorial: “Carotenoids, polyphenols and phytocannabinoids: new perspectives in the prevention of chronic diseases”

**DOI:** 10.3389/fphar.2024.1419129

**Published:** 2024-05-14

**Authors:** Giuseppina Crescente, Stefania Moccia

**Affiliations:** National Research Council, Institute of Food Sciences, Avellino, Italy

**Keywords:** polyphenols, chronic diseases, phytocannabinoids, oxidative stress, antioxidants

Plant-based food matrices are rich in phytochemicals, which are bioactive compounds that can enhance human health and wellbeing when combined with other lifestyle changes. The term “phytochemicals” refers to several classes of compounds including phenolic compounds, alkaloids, carotenoids, and organosulfur compounds (Muscolo et al., 2024). In this regard, polyphenols have been extensively investigated for their ability to protect human health against oxidative damage, which is involved in the development of chronic degenerative diseases, including cardiovascular, neurodegenerative, and metabolic diseases, as well as cancer (Russo et al., 2021). Among these, type 2 diabetes is a metabolic disorder characterized by an alteration in the quantity of insulin or its mechanism of action (American Diabetes Association, 2009). In this context, herbal medicine is gaining attention as a complementary or alternative treatment (Choudhury et al., 2017) and this approach has also been explored by Erukainure et al. analyzing the effect of *C. volubile* leaves against type 2 diabetic rats. In the study, two flavones isolated from the dichloromethane (DCM) fraction of the methanolic extract of *C. volubile* leaves, biochanin, and 5,7,4′-trimethoxykaempferol, demonstrated the highest levels of anti-oxidative activity, β-cell distribution and function, and glucose tolerance. Additionally, treatment with the DCM fraction induced an increase in serum insulin and Ca^2+^ levels, as well as a dose-dependent inhibition of Angiotensin 1 Converting Enzyme (ACE) activity, suggesting potential antihypertensive effects.

Among phytochemicals, quercetin is worth mentioning as a potential candidate for the prevention and management of several diseases (Oboh et al., 2016; Bao et al., 2017). A perspective in the exploitation of quercetin as an anti-calcifier was proposed by Ceccherini et al. who tested a quercetin-enriched extract (purity of 98.1%) in the vascular calcification, an age-related condition characterized by the accumulation of calcium-phosphate salts in the walls of the arteries. The authors highlighted that quercetin was able to lower intracellular calcium levels at all concentrations tested, with the highest reduction (63.11%) at 100 μM. Furthermore, since calcified vascular smooth muscle cells (VSMC) release a considerable amount of inflammatory mediators, some of them have been quantified. At the highest dose tested (100 μM), quercetin reduced IL-6 and TNF-α levels and caspase-1 activity by more than 80% showing its effectiveness in reducing VSMC calcification.

Resveratrol, a polyphenolic phytoalexin, has been successfully studied over the years for its multiple beneficial effects on human health whose antioxidant and anti-inflammatory properties have been associated with the prevention and therapy of cardiovascular diseases, one of the leading causes of death worldwide (Pop et al., 2018; Singh et al., 2019). The review by Godos et al., included in this Research Topic, analyzes unexpected results from randomized clinical trials of resveratrol interventions and discusses how the effects of resveratrol on endothelial and vascular health can be explained through its ability to modulate the response cellular antioxidant and anti-inflammatory pathway. However, the authors conclude that the still inconclusive results strictly depend on the applied concentration and cellular metabolism. Moreover, they interestingly associated inter-individual physiological responses to different compositions of the intestinal microbiota, suggesting a new approach that could explain the role of the microbiota in resveratrol metabolism. Significant changes in the composition of the microbiota involved in resveratrol metabolism are becoming evident: intestinal bacteria such as *Bifidobacteria infantis* and *Lactobacillus acidophilus* contribute to the bioavailability of resveratrol by hydrolyzing the glucoside portions of piceid, which is the precursor to resveratrol, and exposing its aglycones. By glycosylation, resveratrol is converted to piceid, which is then conjugated to the piceid glucuronide and absorbed in its free and conjugated forms. Additionally, intestinal bacteria metabolize piceid to produce dihydropiceid and dihydroresveratrol, and dihydroresveratrol glucuronides are present at higher levels than resveratrol glucuronides and glucosides in human plasma and urine. For this reason, it is more complicated to determine the prebiotic candidate role of resveratrol and its metabolites promoting changes in bacterial composition and whether this interaction contributes to many of its beneficial health effects. Overall, the hypothesis that the cause and development of many diseases could be influenced by the gut microbiota, as a key modulator of disease risk, seems to be plausible (Chaplin et al., 2018). A similar interesting field of investigation is represented by the involvement of the gut microbiota in human physiopathology. A review discussing the impact of gut microbiota on the pathogenesis of diabetic kidney disease (DKD) by Du et al. is included in this Research Topic. In this case, the authors critically analyzed the mechanisms through which natural extracts could exert renoprotective effects by affecting the intestinal flora. A reduction in the production of short-chain fatty acids (SCFA) can be observed in patients, with lower serum levels of acetic acid, propionic acid, and butyric acid. Furthermore, uremic toxins are produced in the plasma as a result of altered protein metabolism in the colon, resulting in chronic renal failure; trimethylamine N-oxide (TMAO) is found at significantly higher levels in patients with chronic renal failure. Here, the authors report that botanical drugs, including *Rheum palmatum* L., *Morus alba* L., *Astragalus mongholicus* Bunge and *Salvia miltiorrhiza* Bunge, as well as resveratrol, punicalagin, curcumin, and fisetin, affect the composition of the intestinal flora, particularly the *Akkermansia*, *Lactobacillus*, and *Bacteroidetes*, SCFA production and restoration of the intestinal barrier; as well as the concentration of uremic toxins (p-cresol sulfate, indole sulfate, TMAO), inflammation and oxidative stress could be reduced in animal models.

To discover new compounds, hemp phytocannabinoids, non-psychoactive and potentially beneficial to human health, are gaining growing interest (Moccia et al., 2020; Nigro et al., 2021; Crescente et al., 2022). A possible application of cannabinoid compounds as adjuvants in the prevention and treatment of chronic diseases has recently been proposed by García-Gutiérrez et al. supported the hypothesis that cannabidiol (CBD) can act as an antidepressant agent in male mice exposed to an unpredictable chronic mild stress (UCMS). The authors demonstrated that CBD reversed behavioral anxiety and hopelessness, and improved cognitive impairment and anhedonia in stressed mice faster than sertraline (STR), an antidepressant drug. Furthermore, CBD normalized all gene expressions of key targets closely related to depression.

To summarise, our Research Topic has aroused great interest as it covers a wide field of research ranging from the possibility of using phytochemical compounds as an aid in the prevention/treatment of conditions underlying chronic diseases to the exploration of the mechanism of action. New horizons have also been opened, including a better understanding of the role of the gut microbiota and the activity of phytochemical metabolites, which suggests a scenario for future investigations. Furthermore, the search for new compounds with interesting properties for human health, including phytocannabinoids, is becoming prevalent. We expect this area to continue to grow to identify potential molecular targets for metabolites and develop innovative functional formulations.
